# Can simulation-based education and precision teaching improve paediatric trainees’ behavioural fluency in performing lumbar puncture? A pilot study

**DOI:** 10.1186/s12909-019-1553-7

**Published:** 2019-05-10

**Authors:** Sinéad Lydon, Bronwyn Reid McDermott, Ethel Ryan, Paul O’Connor, Sharon Dempsey, Chloe Walsh, Dara Byrne

**Affiliations:** 1 School of Medicine, National University of Ireland Galway, Co. Galway, Ireland; 2 Irish Centre for Applied Patient Safety and Simulation, School of Medicine, National University of Ireland Galway, Co. Galway, Ireland; 3 Department of Paediatrics, University Hospital Galway, Co. Galway, Ireland; 4 Discipline of General Practice, School of Medicine, National University of Ireland Galway, Co. Galway, Ireland; 5 Department of Paediatrics, The National Maternity Hospital, Holles Street, Co. Dublin, Ireland

**Keywords:** Simulation training, Education, medical, Spinal puncture, Infant, Patient outcome assessment, Behavioural research

## Abstract

**Background:**

Low levels of success in performing lumbar puncture have been observed among paediatric trainees. This study assessed the efficacy of simulation-based education with frequency building and precision teaching for training lumbar puncture to behavioural fluency.

**Methods:**

The intervention group was assessed at baseline, at the final training trial, in the presence of distraction, and a minimum of one month after the cessation of the intervention in order to ascertain whether behavioural fluency in lumbar puncture was obtained. Subsequently, the performance of this intervention group (10 paediatric senior house officers) was compared to the performance of a comparator group of 10 more senior colleagues (paediatric registrars) who had not received the intervention. Retrospective chart audit was utilised to examine performance in the clinical setting.

**Results:**

Intervention group participants required a mean of 5 trials to achieve fluency. Performance accuracy was significantly higher in the intervention group than the comparator group. Learning was retained at follow-up and persisted during distraction. Retrospective chart audit revealed no significant difference between the performance of the intervention group and a comparator group, comprised of more senior physicians, in the clinical setting, although the interpretation of these analyses are limited by a low number of lumbar punctures performed in the clinical setting.

**Conclusions:**

The programme of simulation-based education with frequency building and precision teaching delivered produced behavioural fluency in lumbar puncture among paediatric trainees. Following the intervention, the performance of these participants was equivalent to, or greater than, that of senior paediatricians. This study supports the need for further research exploring the effectiveness of simulation-based education with precision teaching to train procedural skills to fluency, and the consideration of how best to explore the impact of these on patient outcomes.

**Electronic supplementary material:**

The online version of this article (10.1186/s12909-019-1553-7) contains supplementary material, which is available to authorized users.

## Background

Traumatic infant lumbar puncture is associated with increased treatment costs [1], unnecessary antibiotic use [2], and pain and distress for the infant [3]. Research suggests success (i.e., absence of traumatic lumbar puncture) occurs in 24–54% of performances of lumbar puncture by paediatric residents [4]. While simulation-based education results in improvements in paediatric trainees’ performance of lumbar puncture [5–7], studies have shown that improvements decay over time [5, 6], and/or do not generalise to the clinical setting [8, 9].

One means of addressing the deficits of simulation-based education may be to teach procedural skills to behavioural fluency. Behavioural fluency is the “combination of accuracy plus speed of responding that enables competent individuals to function efficiently and effectively in their natural environment” [10]. Fluent behaviour retains (i.e., persists over time), is stable (i.e., does not deteriorate during distraction), and generalises to novel settings [10, 11].

Precision teaching is an educational framework that facilitates the determination of when fluency is achieved, by ensuring monitoring of a learner’s performance [11]. An intervention incorporating precision teaching typically includes: 1) the establishment of a fluency criterion (i.e., a time in which an expert can comfortably and accurately complete the task); 2) frequency building (i.e., repeated, timed learning trials during which the learner performs the behaviour and receives feedback); 3) use of the standard celeration chart (a semi-logarithmic chart that depicts the frequency of a target behavior and allows for the ascertainment of whether a growth in learning is occurring or whether a change in instructional tactics is required) [12] to monitor progression, and; (4) changing, or supplementing, instructional tactics in instances where a participant is not progressing [13]. Lydon et al. [11] reported that medical students who received simulation-based education with precision teaching to train venepuncture outperformed their untrained peers and hospital doctors. Their learning generalised to the clinical setting, and performance was maintained during distraction and at follow-up.

The current study assessed the efficacy of simulation-based education with frequency building and precision teaching for improving the performance of lumbar puncture among paediatric trainees. Our primary research question was whether behavioural fluency could be established in lumbar puncture. Secondary research questions were whether trained participants performed comparably to more senior colleagues in the simulated setting or in the clinical setting.

## Methods

### Experimental design and setting

**Fig. 1:**
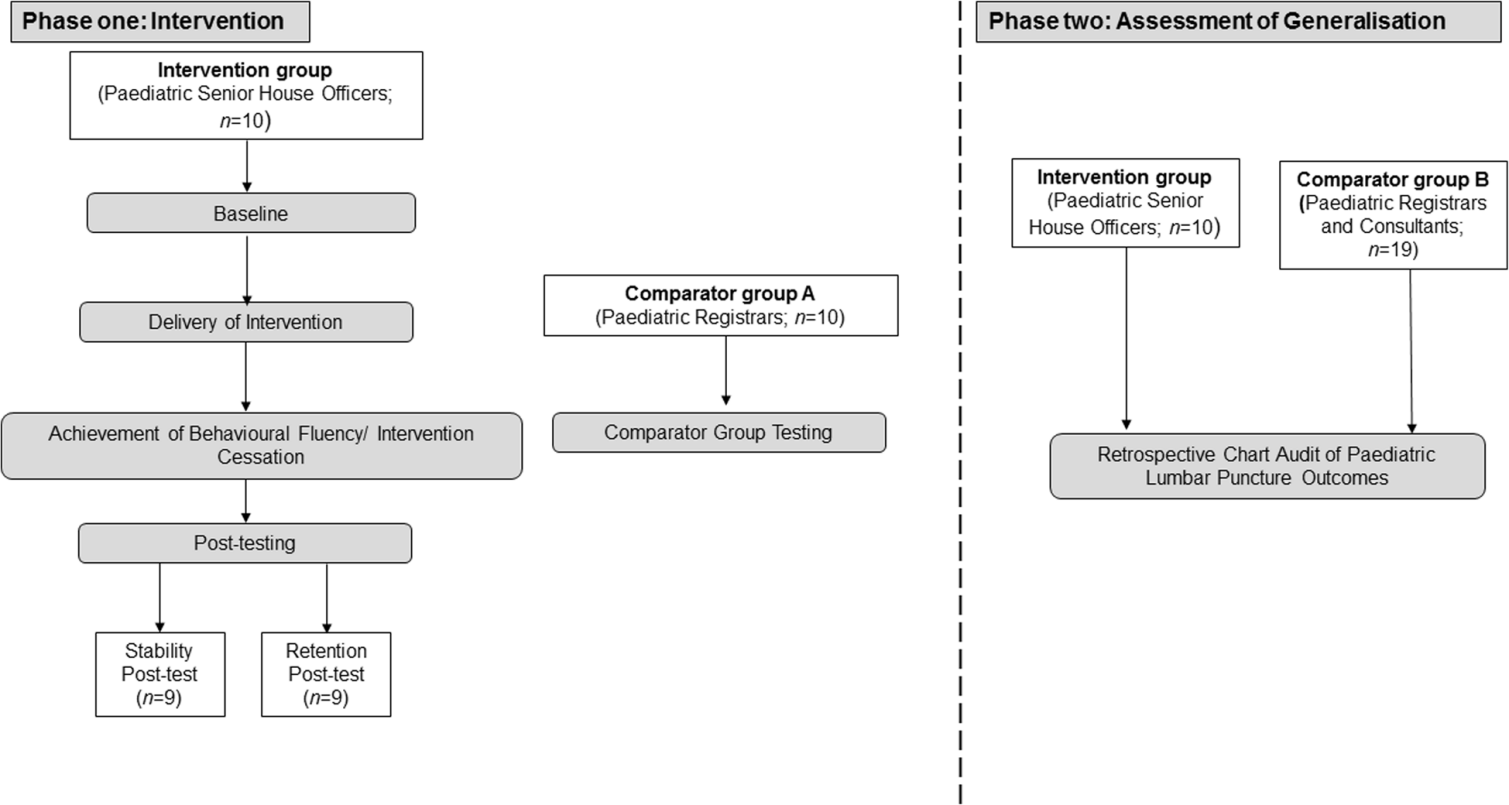
Flow diagram depicting the activities occurring during the Intervention and Assessment of Generalisation phases of this study

This study consisted of an Intervention phase and an Assessment of Generalisation phase (see Fig. 1). Both phases involved between-groups comparisons. In phase one, the performance of the intervention group of 10 participants was compared to comparator group A, which was comprised of 10 paediatric registrars, in the simulated setting. In phase two, the performance of the same intervention group was compared to comparator group B, which comprised of 19 paediatric registrars and consultants, in the clinical setting.

This research was carried out within the Paediatrics Department of one large Irish teaching hospital.

### Ethical approval

Ethical approval was obtained from the Research Ethics Committee of the hospital in which the study was completed.

### Target behaviour

Paediatric lumbar puncture was the target behaviour identified for improvement. Accordingly, the primary dependent variable was percentage accuracy in the performance of lumbar puncture. Determination of the accuracy of the performance of each step of the procedure by trained observers was informed by the lumbar puncture task analysis developed for this study (for further details, see Phase One Procedure subsection below and Additional file 1). The data collected by trained observers was used to inform an overall percentage accuracy score for each trial and the duration of each trial was also recorded.

The trained observers in this study were a simulation technician with expertise in assessment and education via healthcare simulation and a masters-level psychologist with experience in behavioural observation and in the healthcare simulation environment. The observers received comprehensive training in the target behaviour from a Paediatric Consultant. Measures were taken to ensure their observations were calibrated in advance of data collection: they observed and assessed the target behaviour being performed and received feedback on their scoring; they performed the procedure and received feedback; and they were provided with the opportunity to query the purpose of each step of the task analysis and the rationale for conducting it in the manner described.

### Phase one: intervention phase

#### Participants

An a priori power analysis was conducted using G*Power to determine appropriate sample size to power a one-way repeated measures ANOVA with one group assessed across four measurements (i.e., baseline, final training trial, stability trial, and retention trial) of the dependent variable (i.e., percentage accuracy in performance). Alpha was set at .05 and power set at 0.95 for this analysis. The effect size of a similar intervention delivered by Lydon et al. [11] was large (partial η^2^ = .9979). However, with consideration of differences in the sample and target behaviour within Lydon et al. [11] and the current study, a more conservative estimate of a large effect size was entered into G*Power (partial η^2^ = .25). An intervention group size of 8 was found to be sufficient to power a one-way repeated measures ANOVA with four measurement points.

Convenience sampling was used to recruit 20 paediatric trainees. Convenience sampling, a form of non-random sampling, involves the recruitment of participants who meet the study’s inclusion criteria based primarily upon their accessibility or availability to the researchers [14]. In this case, participants were recruited from the hospital with which the researchers were affiliated. Convenience sampling is commonly used in quantitative research studies, but may introduce bias (e.g., through self-selection) and impact the representativeness of the sample of participants [15]. Ten of the recruited participants were senior house officers (equivalent to a junior resident; six females, four males) who took part in the intervention group. Mean age was 28.1 years (SD = 2.9) and mean number of years in clinical practice was 3.3 (SD = 2.5).

Comparator group A consisted of 10 registrars (equivalent to a senior resident; three females, seven males). The use of a more senior comparator group in healthcare simulation research studies is not uncommon [11, 16, 17], and allows for the consideration of the impact of the intervention as compared to the impact of increased clinical experience. Mean age in comparator group A was 32.8 years (SD = 6) and mean number of years in clinical practice was 7.9 (SD = 3.2).

#### Materials

All equipment used was the same as that available for performing lumbar puncture in the Paediatrics Department (for further detail, see Additional file 1).

The simulator used was the task trainer LumbarPunctureBaby supplied by Simulab, which replicates the anatomy of a neonate. The body of the task trainer contains an iliac crest and umbilicus and the model is flexible to allow for positioning. The simulator is fitted with a replaceable lumbar block, which contains two fluid tubes- a spinal cord and the epidural venous plexus- which were filled with simulated cerebrospinal fluid and blood.

In addition, each observer had data collection sheets, a timer and a copy of the task analysis (described below).

#### Procedure

##### Preparation

Precision teaching requires the ‘pinpointing’ of the target behaviour. [13] In order to “pinpoint” a behaviour, an objective written definition of the observable, physical movements of a behaviour must be developed [13]. Lumbar puncture was pinpointed by developing a task analysis (see Additional file 1) that listed the discrete steps involved in performing the behaviour, and provided clear explanations of the physical movements and materials required to perform each step. The task analysis was informed by reviewing relevant clinical practice guidelines, the input of a paediatric consultant with over 20 years of clinical experience, and feedback from the hospital’s microbiology department, a paediatric nurse, and an infection control nurse. The face validity of the task analysis was confirmed by having two senior academic physicians, with expertise in the design and delivery of simulation based education for procedural skills training, review the document and provide feedback.

Following this, the fluency criterion was established. A paediatric consultant carried out a lumbar puncture on three occasions using the LumbarPunctureBaby. Each performance was 100% accurate, and the median duration of these performances was used to set the fluency criterion. The fluency criterion was set at 19 mins 53 s and below, requiring participants to complete the task with 100% accuracy without exceeding the expert’s median duration by more than 10%.

### Evaluation of intervention

#### Baseline *(Intervention Group only)*

Participants in the intervention group attended the simulation laboratory for baseline testing. The participants were oriented to the simulator and its “anatomy” by one of the two trained observers. All materials for carrying out the procedure were provided to the participants. The participants were instructed to begin the lumbar puncture procedure at the point of having obtained consent. Participants were told they would end the procedure following the writing-up of the procedure in the clinical note. Following these preparatory instructions, the participants were asked to carry out lumbar puncture ‘to the best of their ability’ and informed that the accuracy and duration of their performance would be assessed by one of the two trained observers. Although the participants were not provided with corrective feedback upon completion of the trial, they were provided with the task analysis.

#### Delivery of intervention *(Intervention Group only)*

An overview of the intervention and the criterion for being deemed fluent (i.e., 100% accuracy within 19 mins and 53 s achieved in two consecutive trials) was provided to participants.

Each performance of lumbar puncture was referred to as a frequency building trial. An “assistant” was present for each trial who played the role of a nurse and could be directed by participants. All trials were observed and timed by one of the two trained observers. The recording sheet required observers to provide a binary indication of whether each step was completed accurately or inaccurately. Written notes were made of any inaccuracies in the performance of a step. Upon conclusion of the trial, the observer provided the participant with corrective feedback. Specific, detailed corrective feedback was delivered by one of the trained observers and focused both on the accuracy of the performance and its pace. The observer indicated the total number of steps performed correctly, detailed any steps that had been performed incorrectly, and demonstrated the correct performance of these steps on the simulator if desired. Pace was considered within feedback with reference to the time associated with the fluency criterion. Participants were also given the opportunity to practice any steps completed incorrectly.

Throughout the intervention phase, participants’ data (steps performed correctly, steps performed incorrectly, and duration of performance) were used to produce standard celeration charts that were produced using Chartlytics Software (www.chartlytics.com). Standard celeration charts have been widely used to record behaviour, to clarify whether learners are progressing under particular instructional conditions, and facilitate instructor decision making relating to any need for supplemental instructional tactics [12]. In this study, the charts were reviewed by researchers and did not indicate a need for supplemental instruction for any participants. However, it was noted by trained observers that although all participants were progressing, they were consistently being marked as inaccurate on Step 16 “Instructs assistant to position child in correct manner during the procedure”. Participants were typically failing to provide adequate instruction to the assistant and to recognise correct positioning. This led to subsequent difficulty with the angle of needle insertion and resulted in unsuccessful taps. Participants were given a “booster session” in which a consultant paediatrician demonstrated how the infant should be correctly positioned and participants had opportunities to position the infant, insert the needle, and receive feedback.

#### Post-testing *(Intervention Group only)*

Two post-tests were conducted following intervention cessation.

##### Retention

The retention post-test was conducted in the same manner as baseline testing but took place a minimum of one month post-intervention (M = 84.2 days, SD = 46.6).

##### Stability

The stability of behaviour was assessed by examining performance of lumbar puncture in the presence of distraction. Communication irrelevant to an ongoing clinical task is a key distractor in clinical practice [18]. Therefore, we utilised a series of interrupting questions that were not related to the ongoing task (e.g., ‘tell me about the most recent book that you read’) as the distraction. Delivery of the interrupting questions was standardised with regards to the ordering and timing of questions. A new question was delivered every minute and participants were prompted to provide further information if their answers were brief.

### Comparator group testing

The 10 paediatric registrars in comparator group A visited the simulation laboratory individually. They were asked to perform lumbar puncture in test conditions equivalent to those used for baseline testing in the intervention group. Accuracy and duration of performance were recorded.

#### Analysis

Preliminary analysis revealed that the data collected were not normally distributed. As a result, non-parametric statistical analysis was conducted. First, a Friedman’s ANOVA was conducted to compare the performance of the intervention group at baseline, final intervention trial, stability post-test, and retention post-test. Post hoc testing was conducted using a series of Wilcoxon signed-rank tests, and alpha was corrected to .008 to account for these multiple comparisons.

The comparison of performance accuracy of the intervention and comparator group A was assessed using a Mann-Whitney test (the non-parametric equivalent of an independent samples t-test). The Mann-Whitney test allows for the comparison of non-normally distributed data collected from two different groups.

### Phase two: assessment of generalisation phase

#### Participants

During this phase, the performance of the intervention group from Phase 1 was compared to comparator group B, a group of more senior doctors; all paediatric registrars, specialist registrars, and consultants working in the Paediatrics Department. There were a total of 10 registrars (4 female, 6 male), 2 specialist registrars (both females) and 7 consultants (4 female, 3 male) practicing during the chart audit period.

#### Procedure

A retrospective chart audit was conducted of the charts of patients under 12 months who had undergone lumbar puncture in the 12 month period beginning July 2016. The 12 month age cut-off was used as the procedure for performing lumbar puncture is considered to be the same from 0 to 12 months, but may differ beyond this. Charts were excluded if the lumbar puncture was carried out by an individual other than an intervention group participant, paediatric registrar, paediatric specialist registrar, or consultant, or if it was not possible to determine who performed the procedure. Retrospective chart audit has been used previously to assess lumbar puncture performance among paediatric trainees [19]. For each chart, the individual responsible for carrying out the procedure was recorded (i.e., intervention group participant or paediatric registrar/specialist registrar/consultant) along with the lumbar puncture outcome. Ascertainment of lumbar puncture outcome was based solely on objective data provided by the hospital’s microbiology laboratory. Previous research has reported a variety of definitions for traumatic lumbar puncture, with the amount of red blood cells considered indicative of traumatic lumbar puncture ranging from 400 to 10,000 across studies [20]. In this study, the outcome of lumbar puncture was recorded as successful in instances in which laboratory data indicated that at least one sample contained < 1000 red blood cells [6, 8]. All other instances were recorded as traumatic.

#### Analysis

A Risk Ratio was calculated to compare the probability of a traumatic lumbar puncture between the two groups.

## Results

### Phase one: intervention

#### Intervention group performance

Participants required a mean of five trials (SD = 1.2) to achieve fluency and mean total training time was 95 mins, 24 s (SD = 31 mins, 6 s). A sample standard celeration chart generated is available in Additional file 2.

**Fig. 2:**
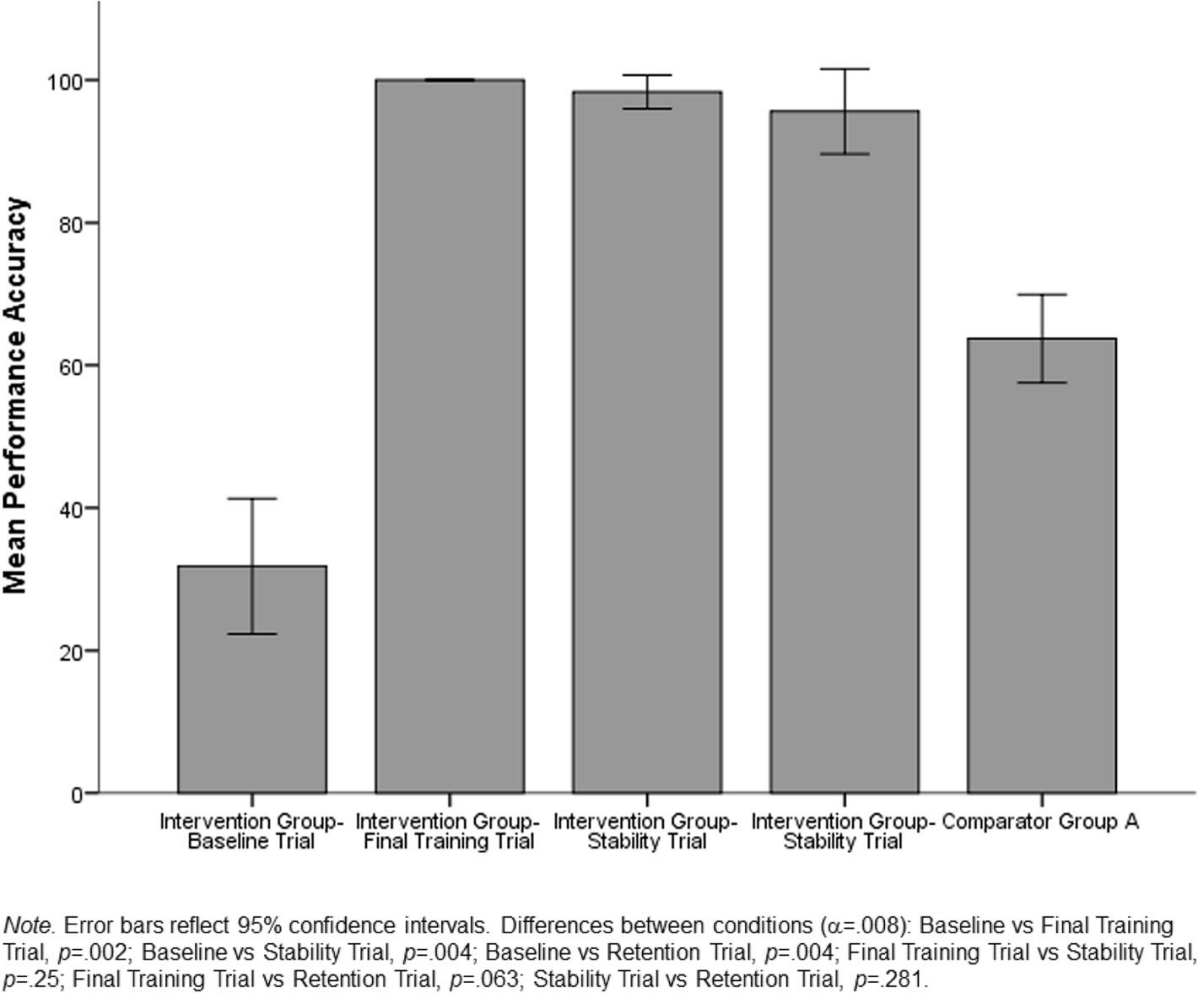
Mean performance accuracy for intervention group and comparator group A across study measurement timepoints

Performance of the members of the intervention group is depicted in Fig. 2. A Friedman’s ANOVA indicated that there was a significant difference in performance across conditions, χ^2^(3) = 22.66, *p* < .001. Post hoc testing was conducted with a Bonferroni correction applied to control for the use of multiple comparisons and α set at .008. Post hoc testing revealed significantly lower accuracy at baseline (*Median* = 33.33) than at the final intervention trial (*Median* = 100), the stability trial (*Median* = 96.96), and the final retention trial (*Median* = 100). No significant differences in accuracy were observed between the final intervention trial, the stability trial, and the retention trial.

#### Comparison with comparator group A

Figure 2 illustrates the differences between mean performance accuracy of comparator group A and the intervention group’s performance across the timepoints. A Mann-Whitney test revealed that intervention group participants were performing lumbar puncture with significantly more accuracy (*Median = 100*) post-intervention than were the paediatric registrars in comparator group A (*Median* = 63.64), U = 0, *p* < .001.

### Phase two: assessment of generalisation

Table 1 presents the rates of successful and traumatic lumbar punctures by group, along with the outcomes of the Risk Ratio analysis. The Risk Ratio analysis revealed no statistically significant difference in the frequency of traumatic lumbar puncture between the two groups.

**Table 1:** Comparison of rates of successful and traumatic lumbar punctures by group and risk ratio analysis outcomes

	Intervention Group (Pediatric Senior House Officers; *n* = 10)	Comparator Group B (Pediatric Registrars and Consultants; *n* = 19)
Total number of LPs performed:	*n* = 12	*n* = 45
Number of successful LPs:	*n* = 11; 91.7%	*n* = 25; 55.6%
Number of traumatic LPs:	*n* = 1; 8.3%	*n* = 20; 44.4%
Risk Ratio Analysis:	Relative risk = 0.19 95% CI = .03 to 1.3 *z* = 1.7 *p* = .09	

*Note.* LP = Lumbar Puncture

## Discussion

This study evaluated the effects of simulation with frequency building and precision teaching for improving the performance of lumbar puncture among paediatric trainees. Three impacts of the intervention were observed: 1) the intervention group’s performance improved significantly from baseline and improvements in performance persisted over time (retention) and during distraction (stability); 2) post-intervention the intervention group significantly outperformed more senior paediatric trainees in the simulated setting, and; 3) statistically equivalent rates of bloody taps between the intervention group and the more clinically experienced comparator group B which was comprised of more senior paediatric physicians. Most importantly, participants achieved behavioural fluency in lumbar puncture and subsequently performed at the level of more clinically experienced colleagues.

Members of the intervention group achieved fluency in an average of five trials and 95 min of training. Two key characteristics of behavioural fluency- retention and stability- were also evident. Retention was evident when intervention group participants’ high level of accuracy persisted at follow-up. Stability was evident through participants’ continued high level of accuracy in spite ongoing distraction [18]. These data are important given that previous research has noted that improvements in the performance of lumbar puncture following simulation-based education fail to retain [5, 6]. Learning also appeared to generalise to the hospital setting, where the outcomes of lumbar punctures performed by participants were not distinguishable from those performed by physicians of a higher grade and greater clinical experience.

It is not possible to state conclusively that simulation-based education with frequency building and precision teaching leads to better outcomes than would be achieved if trainees were appropriately supervised by experienced consultants while performing the procedure in the actual clinical setting. However, our data showed significantly superior performance of the intervention group as compared to more senior colleagues in the simulated setting (comparator group A). There is substantial value in the intervention delivered that may not be garnered through increased clinical experience alone. The ability to perform procedural skills to fluency ensures clinical competence and decreased clinical errors leading to greater patient safety [21]. Future research should further evaluate the potential for this type of intervention to contribute to improvements in procedural skills that may directly impact patient outcomes. Studies that consider how incorporating technological innovations (e.g., ultrasound guided lumbar puncture [22]) may expedite the achievement of behavioural fluency, and/or improve training outcomes, would be of particular interest.

### Considerations for future research and practice

There was no statistically significant difference between the outcomes of lumbar punctures (based solely on laboratory red blood cell count) performed by the intervention group and a comparator group B. This comparator group comprised of more senior physicians, who were also likely more clinically experienced (although data was not collected on comparator group B’s prior experience in lumbar puncture). Consideration of the complexity of the individual lumbar punctures performed by both groups would have been useful to elucidate any differences in ability across the two groups. However, this was not possible through the retrospective chart audit process given the reliance on clinical notes. The success rate observed among the intervention group (> 90%) does compare favourably with success rates of 24–54% that have been observed among paediatric residents elsewhere [4]. Previous research has also highlighted a low number of clinical lumbar punctures as an impediment to ascertaining an intervention’s impact [20]. Such data relating to limited clinical opportunities to perform procedural skills indicate the importance of delivering empirically-supported simulation-based education to train key skills such as lumbar puncture. Paediatric trainees also report a low preparedness for other essential skills, such as the management of cardiopulmonary arrests [23]. Future research must consider how best to assess generalisation when clinical opportunities may be few.

The benefits of peer tutoring are well-established [24]. Lydon et al. [11] reported that their participants were able to accurately assess their peers’ performances. Ensuring that participants develop the ability to accurately assess performance and provide corrective feedback may be important. Previous research [25] has suggested that individuals taught procedural skills using mastery training become better teachers. Future research examining the use of precision teaching within simulation-based education should endeavour to incorporate peer tutoring and to explore the impact of this.

Future research in this area may benefit from including an economic evaluation of the intervention. Economic evaluation is widely used in healthcare, but uncommon in medical education [26]. Simulation-based education is recognised as a resource-intensive process [27] and there is a need to report whether it produces improved performance and/or patient outcomes [26]. Cost analyses are therefore crucial in supporting the utility of simulation-based education [26].

### Limitations

There are a number of limitations to this study. First, the small sample size and reliance on non-random sampling and group assignment may be criticised. However, an a priori power analysis indicated that an intervention group comprised of 10 participants offered adequate power for the analysis, and there are important ethical considerations surrounding the recruitment of participants beyond what is necessary to adequately and appropriately power a study [28, 29]. Second, the setting of the fluency criterion on the basis of the performance of just one consultant may be criticised. The fluency criterion relates to the performance of the procedure as per the task analysis with complete accuracy and the time this requires. Therefore, it was not suitable to use other estimates within the literature as these procedures were not performed in accordance with the specific task analysis. However, future research could establish the fluency criterion on the basis of the observation of multiple experts. Third, the lack of testing of the comparator group A at baseline may also be considered a weakness, although previous research studies have employed a similar design [11, 30]. There is a need to utilise stronger experimental designs in the evaluation of simulation-based education incorporating precision teaching. However, it may be challenging to implement such designs in the context of the clinical environment. Finally, the assessment of the generalisation of the learning to the clinical setting was limited by the use of retrospective, rather than prospective, chart audit and the relatively low number of lumbar punctures performed within the Paediatric Department. Future research should rely on prospective data collection which would allow greater insight into each clinical performance of the targeted skill and the outcomes and a better understanding of generalisation of the targeted behaviour.

## Conclusions

Simulation-based education with frequency building and precision teaching produced behavioural fluency in lumbar puncture among paediatric trainees. The intervention yielded a level of performance equivalent to, or greater than that, of senior paediatricians and learning was observed to retain, be stable, and appeared to generalise to the real hospital environment. These data support the continued exploration of fluency training and precision teaching within medical education.

## Additional files

Additional file 1: Paediatric Lumbar Puncture Task Analysis. (PDF 125 kb)

Additional file 2: Sample of a completed standard celeration chart for a participant in the intervention group. (PPTX 151 kb)

## Abbreviations

ANOVA: Analysis of variance
M: Mean
p: probability
SD: Standard deviation
