# Supplementary material for: Can simulation-based education and precision teaching improve paediatric trainees’ behavioural fluency in performing lumbar puncture? A pilot study
Source: BMC Med Educ. 2019 May 10;19:138. doi: 10.1186/s12909-019-1553-7 (PMC6511218; doi:10.1186/s12909-019-1553-7)
Supplement: Supplementary file 1 — Paediatric Lumbar Puncture Task Analysis. (PDF 125 kb) [file 12909_2019_1553_MOESM1_ESM.pdf]

Paediatric Lumbar Puncture Task Analysis

ICAPSS Irish Centre for Applied Patient Safety & Simulation

Task	Operational Definition
**1. Applies mask**	Apply mask over nose and mouth and tie both sets of ribbons behind head.
**2. Applies cap**	Apply cap so that elastic is at the neck and ensure all hair is secured inside.
**3. Performs hand hygiene using alcohol gel**	Apply alcohol gel to the palm of one hand and rub both palms together so that gel is distributed.<br>Rub the back of each hand with the palm of the other hand with fingers interlaced.<br>Rub palm to palm with fingers interlaced.<br>Rub with back of fingers to opposing palms with fingers interlocked. Enclose the thumb of one hand within a fist of the other and rotate for both hands consecutively.<br>Rub tips of fingers in opposite palm in a circular motion.<br>Rub each wrist with opposite hand using a circular motion.
**4. Cleans trolley with 70% alcohol wipes**	Clean the top of the trolley thoroughly using 70% alcohol wipes.
**5. Places sterile drape on trolley**	Open one sterile drape and drop onto the centre of the trolley without touching.<br>Open out sterile drape and use to cover the surface of the trolley.
**6. Opens dressing pack and places on trolley**	Open the sterile dressing pack away from the trolley and drop onto the centre of the trolley without touching.
**7. Opens out dressing pack maintaining aseptic conditions**	Open out the dressing pack by touching only the corners of the pack. Aseptic conditions must be maintained. If necessary, the yellow waste bag inside of the dressing pack may be used to temporarily cover your hand and rearrange the contents of the pack.
**8. Pours cleansing solution into tray**	Pour cleansing solution into the 2 small compartments in the tray ensuring the bottle does not make contact with the sterile field.
**9. Places all equipment on sterile field maintaining aseptic conditions**	All equipment is opened away from the sterile field and then dropped into the sterile field without touching. Open spinal needle and place onto the sterile field. Open gloves and place onto the sterile field. Open gown and second drape and place on sterile field. Open waterproof dressing and place on the sterile field. Spray-on dressing must not be placed on the sterile field.
**10. Labels collection bottles (1, 2, 3.)**	Label collection bottles (both bottle and lid) with numbers 1, 2, 3 or request that your assistant do this.
**11. Aligns bottles and places away from the sterile field**	Align the bottles according to their numbers and place away from the sterile field.
**12. Performs scrub**	Use Chlorhexidine scrub (Hibiscrub) and running water to complete surgical hand

	hygiene (this should last minimum of 1.5 minutes).<br><br>Turn on taps and adjust temperature. Wet arms and forearms. Turn off taps. Apply 3 pumps (5 mls) of antiseptic agent to palms and forearms to 2.5cm above the elbow for 30 seconds. Turn on taps using non-touch technique (i.e. using elbows). Rinse with water from fingertips to elbows.<br><br>Apply 3 pumps (5 mls) of antiseptic agent to palms and forearms, as before, and rub both palms together so that the Hibiscrub is distributed.<br>Rub the back of each hand with the palm of the other hand with fingers interlaced.<br>Rub palm to palm with fingers interlaced.<br>Rub with back of fingers to opposing palms with fingers interlocked. Enclose the thumb of one hand within a fist of the other and rotate for both hands consecutively.<br>Rub tips of fingers in opposite palm in a circular motion.<br>Rub each wrist and forearm with opposite hands using a circular motion. Rinse from fingertips to elbows.<br><br>Turn off taps with elbows. Allow excess water to drain from elbows into sink.<br>Walk to sterile field and dry hands with sterile towels, using one towel per hand.<br>Dry hand with one half of paper towel, fold and dry arm from wrist to elbow (patting motion). Repeat for other hand. Discard paper towels.
**13. Applies sterile gown**	Apply sterile gown. Keep the cuffs of the gown covering hands as you approach the sterile field.
**14. Applies sterile gloves**	Apply sterile gloves. Open out the wrapping of the gloves using only the edges and turn the gloves so that the left and right symbols are visible. Begin by facing the palm of one hand upwards and use your other hand to grasp only the upturned cuff of the glove and pull it securely over your hand. Using your gloved hand, slip your fingers into the inside fold of the opposite glove and avoid touching the outer part of the cuff as you pass your hand into the glove. Fix both gloves so that they fit comfortably and pull up cuffs without touching your skin.
**15. Places sterile drape under child**	Pick up sterile drape from the sterile field and place under the child with the help of your assistant.
**16. Instructs assistant to position child in correct manner during the procedure**	Ensure the infant is positioned at a height that is comfortable for you to perform the procedure. The infant should be placed at the edge of the surface he/she is on with spine flexed and parallel to the surface. It is essential that the assistant maintain this position and that length of the baby is parallel to the table.
**17. Paints/cleans the area**	Using the Chlorhexidine solution, forceps and cotton ball, apply the solution to the child's back starting at the centre of the insertion point and working outwards in a circular motion. Clean in a circular motion from inside to out from the proposed injection site. Repeat three times, making sure a large enough area is cleaned.

	Discard cotton balls between each clean.
**18. Allows to dry for 30 seconds**	Allow cleansing solution to dry for 30 seconds.
**19. Checks spinal needle stylet**	Check spinal needle stylet removes easily by taking it out from the sheath and then replace again and ensure it is locked down.
**20. Identifies puncture site at A.S.I.S.**	Identify the anterior superior iliac spine of the iliac crest on the infant. Place one finger on the crest and trace your fingers down to the corresponding intervertebral space. This leads you to the site of puncture in the midline at approx. the L3 or L4 level which is below the level of the spinal cord.
**21. Stabilizes child by placing hand on sacrum**	Place hand on sacrum to stabilise the baby and aim towards the umbilicus.
**22. Inserts spinal needle bevel up**	Face the bevel upwards. As you insert the spinal needle, you may or may not feel a change in resistance called a "pop" when the needle penetrates the dura. If you do not feel a pop periodically withdraw the stylet and check if there is CSF flow. Remove the stylet and the CSF should flow.
**23. If CSF does not flow...**	If there is no flow, replace the stylet and advance the needle slowly and gently. If necessary, withdraw the needle completely and try again using a second spinal needle.
**24. Collects CSF using sterile technique**	Instruct your second assistant to open the lids of the specimen collection bottles and place them under the needle so that CSF may be collected. Ensure the sterile field is not contaminated. If there is no second assistant available then you must prepare the collection bottles and collect the CSF yourself. Collect CSF in 3 bottles in the correct order (1, 2 , 3.) Obtain 10 drops of CSF per bottle. Close lids of collection bottles securely.
**25. Replaces stylet and remove needle**	Reinsert the stylet into the spinal needle before removing the needle from the child's back.
**26. Disposes of sharps correctly**	Dispose of all sharps correctly by immediately placing in sharps bin.
**27. Applies pressure to puncture site with gauze**	Obtain haemostasis by applying pressure on site with gauze, ensuring bleeding has stopped before moving on to next instruction
**28. Ensures bleeding has stopped and apply dressing**	Ensure bleeding has stopped. Apply spray-on dressing to the puncture site and surrounding area. Remove the backing from a waterproof surgical dressing and apply over the puncture site as a secondary dressing.
**29. Disposes of all clinical waste correctly**	Clinical waste must be disposed of in a yellow clinical waste bag. Gather up the dressing pack and all contents and your gloves and dispose.

**30. Performs hand hygiene using alcohol gel**	Apply alcohol gel to the palm of one hand and rub both palms together so that gel is distributed.<br>Rub the back of each hand with the palm of the other hand with fingers interlaced.<br>Rub palm to palm with fingers interlaced.<br>Rub with back of fingers to opposing palms with fingers interlocked. Enclose the thumb of one hand within a fist of the other and rotate for both hands consecutively.<br>Rub tips of fingers in opposite palm in a circular motion.<br>Rub each wrist with opposite hand using a circular motion.
**31. Labels collection bottles and place in laboratory bag**	Clearly label each bottle with a patient sticker and place in a labelled bag. Send to laboratory for cell count, biochemistry, micro.
**32. Contacts porter and lab**	Phone the porter to collect the lab request bag and contact the lab to let them know the sample is coming or communicate to the nurse that this must be done.
**33. Documents the procedure in the clinical notes (see example)**	Record date and time. Record having obtained informed consent. Record procedure steps. Record outcomes (first pass, bloody fluid, baby moving limbs post procedure etc.) Record what the fluid is being analysed for (which labs it is being sent to). Record that parents have been informed of outcomes. Sign and write your own details (name, MC number and bleep number if relevant.)
