# Supplementary material for: Can simulation-based education and precision teaching improve paediatric trainees’ behavioural fluency in performing lumbar puncture? A pilot study
Source: BMC Med Educ. 2019 May 10;19:138. doi: 10.1186/s12909-019-1553-7 (PMC6511218; doi:10.1186/s12909-019-1553-7)
Supplement: Supplementary file 2 — Sample of a completed standard celeration chart for a participant in the intervention group. (PPTX 151 kb) [file 12909_2019_1553_MOESM2_ESM.pptx]

## Slide 1
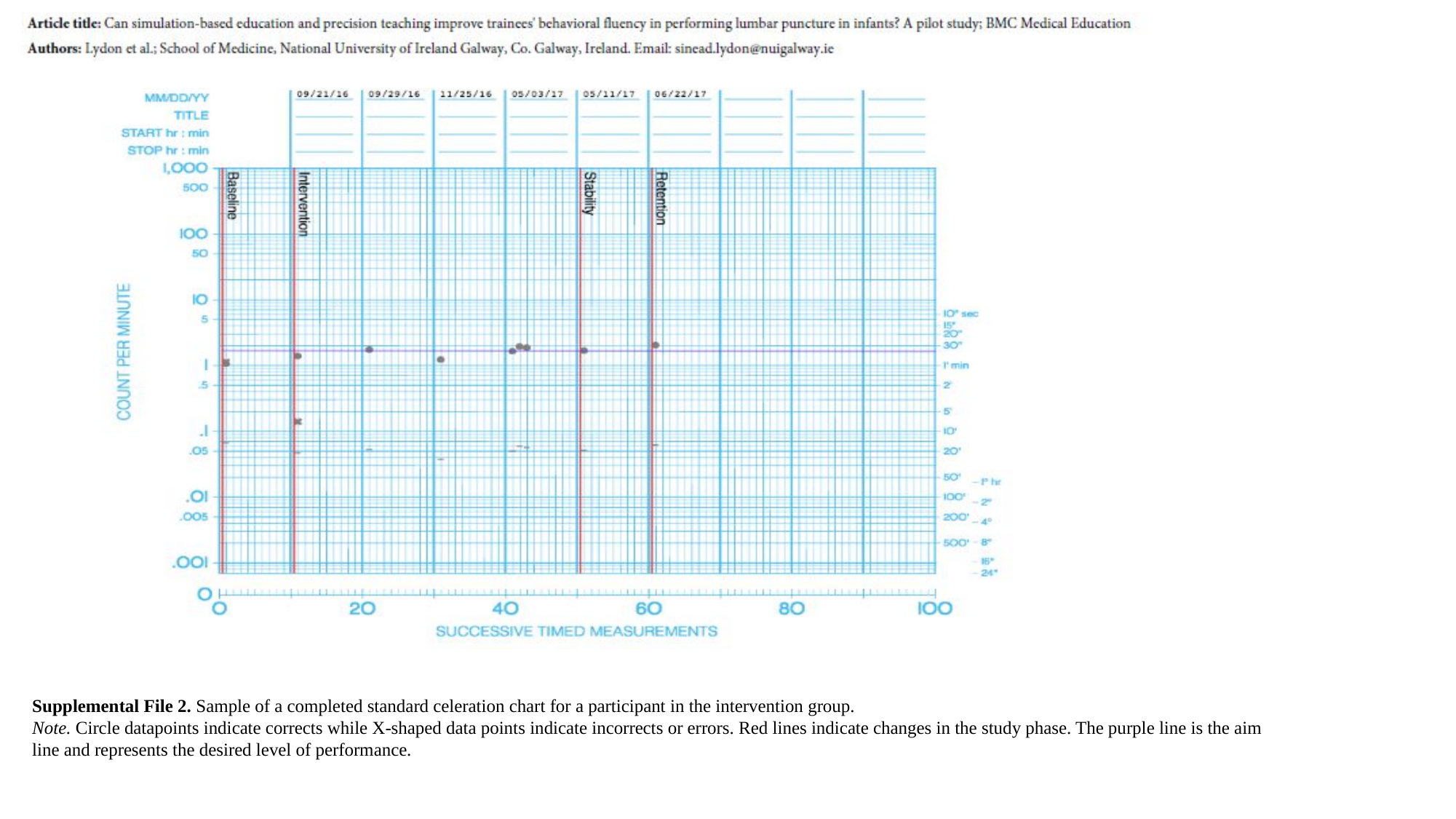

Supplemental File 2. Sample of a completed standard celeration chart for a participant in the intervention group.
Note. Circle datapoints indicate corrects while X-shaped data points indicate incorrects or errors. Red lines indicate changes in the study phase. The purple line is the aim line and represents the desired level of performance.
